# Plethysmographic Peripheral Perfusion Index: Could It Be a New Vital Sign?

**DOI:** 10.3389/fmed.2021.651909

**Published:** 2021-10-01

**Authors:** Mamdouh M. Elshal, Ahmed M. Hasanin, Maha Mostafa, Reham M. Gamal

**Affiliations:** ^1^Department of Anesthesia and Critical Care Medicine, National Cancer Institute, Cairo University, Cairo, Egypt; ^2^Department of Anesthesia and Critical Care Medicine, Cairo University, Cairo, Egypt

**Keywords:** peripheral perfusion index, plethysmography, critically ill, anesthesia, shock

## Abstract

The plethysmographic peripheral perfusion index (PPI) is a very useful parameter with various emerging utilities in medical practice. The PPI represents the ratio between pulsatile and non-pulsatile portions in peripheral circulation and is mainly affected by two main determinants: cardiac output and balance between sympathetic and parasympathetic nervous systems. The PPI decreases in cases of sympathetic predominance and/or low cardiac output states; therefore, it is a useful predictor of patient outcomes in critical care units. The PPI could be a surrogate for cardiac output in tests for fluid responsiveness, as an objective measure of pain especially in un-cooperative patients, and as a predictor of successful weaning from mechanical ventilation. The PPI is simple to measure, easy to interpret, and has continuously displayed variables, making it a convenient parameter for detecting the adequacy of blood flow and sympathetic-parasympathetic balance.

## Introduction and Aim of the Work

The pulse oximeter is a basic monitor in medical practice with an essential role to evaluate the peripheral oxygen saturation and heart rate using plethysmography technology. Pulse oximetry-derived peripheral perfusion index (PPI) is another variable that is measured by pulse oximeters using relatively advanced technology, namely, co-oximetry. The PPI represents the ratio between the portions of the blood in the peripheral tissue, namely, the pulsatile and the non-pulsatile blood flow. The PPI is measured by different types of monitors (e.g., Masimo Corporation, Irvine, CA, USA).

Peripheral perfusion index values depend on the blood flow in the peripheral circulation and the vascular tone; thus, it reflects two main determinants, which are the cardiac output and the balance between the sympathetic and the parasympathetic nervous systems. Being a representative of those two major hemodynamic parameters, the PPI could provide very useful information during the initial evaluation, risk stratification, and follow-up. The normal value of PPI was suggested to range between 0.2 and 20%; however, an observational study showed a median (quartiles) normal value of PPI of 4.3 (2.9–6.2) (1). The values of PPI are highly skewed and therefore, it is more commonly used for follow-up in comparison to the baseline readings of each individual (2).

This review is aimed to clarify the different benefits of monitoring the PPI and the pitfalls and the limitations of its measurements. An overview of its uses in emergency departments, intensive care units, and operating theaters will be also provided.

## PPI as Severity Indicator in Critically Ill Patients

Being a representative of sympathetic-parasympathetic balance, the PPI decreases in conditions with sympathetic overactivity, which is predominant in critical illness and circulatory failure (3). The PPI decreases when there is sympathetic stimulation; therefore, although septic shock is characterized by vasodilatory and distributive nature, this vasodilatation does not apply to peripheral blood vessels (2). Thus, the more the more severe the shock, the lower the PPI is (4). Low PPI predicted poor outcomes in various groups of critically ill patients, such as patients with sepsis (5) and post-cardiac arrest patients (1). In patients with severe sepsis or septic shock, a PPI < 0.3 can predict the need for vasopressor therapy. Furthermore, a PPI < 0.2 can predict patient mortality (5, 6). In patients with post-return of spontaneous circulation (ROSC) after an out-of-hospital arrest, the mean PPI in the first 30 min was independently associated with patient outcomes, i.e., 30-day mortality or poor neurologic outcome (1). Patients with the lower tertile PPI showed double-fold mortality compared to those with higher PPI values (1). As higher PPI reflects better peripheral tissue perfusion, it is linked to good patient outcomes.

Recent evidence also suggests that PPI could be used to guide and titrate vasopressor pressor therapy (7).

## PPI as a Guide for Fluid Therapy

As PPI is affected by both cardiac output and vasomotor tone, PPI could be an indicator of the cardiac output in case there is no change in the sympathetic activity. Furthermore, PPI showed a good ability to detect changes in the cardiac output in patients with septic shock (8).

Hence, PPI was used in fluid responsiveness tests as a surrogate for cardiac output in various maneuvers (9). An increase of 9% in the PPI after a passive leg raising test (10) and 2.5% after end-expiratory occlusion (11) test could predict fluid responsiveness with fair predictive value. An increase of 5% in the PPI after a 200-ml fluid bolus can predict fluid responsiveness in patients with septic shock (12). A decrease of 26% in the PPI after the lung recruitment maneuver can also predict fluid responsiveness in the operating room (13). This use represents a great achievement in guiding fluid therapy in settings where a cardiac output monitor is not available (13). It should be noted that the value of PPI in different tests of fluid responsiveness is more prominent in the positive predictive value than the negative predictive value; therefore, the increase in the PPI with preload challenge can detect responders while the failure of the PPI to increase does not confidently rule out fluid responsiveness.

## PPI as an Objective Measure for Pain

Pain assessment usually relies on subjective scores, which require patient cooperation. Therefore, evaluation of pain in un-cooperative patients, such as critically ill patients, is usually challenging and requires a cumbersome scoring system. Finally, there are no tools to provide real-time measurement of pain. Various studies used the relation between PPI and sympathetic activity as an indirect method for pain evaluation (14, 15). There was a correlation between the change in PPI and the change in the behavioral pain scale in non-intubated patients (15). A decrease in the PPI value by 0.7 could accurately detect a 3-point change in the behavioral pain scale in non-intubated patients (15).

## PPI in the Operating Room

Various uses were reported for the PPI in the operating room. Some uses of the PPI in the operating room rely on its relationship with the vasomotor tone, such as discrimination of failed and successful peripheral nerve blocks (16) and neuraxial blocks (17). An increase in the PPI value by 40% from the baseline value can detect successful supraclavicular brachial plexus block (16). Other authors reported that the PPI can trace the changes in central hemodynamics under general anesthesia where the sympathetic activity is commonly reduced (18). PPI was also found a predictor of postoperative complications after major surgeries (19, 20). Furthermore, intraoperative PPI could be used to tailor the use of vasoactive drugs to provide safe hypotensive anesthesia (21).

## Other Uses of PPI in Critically Ill Patients

In critically ill patients, the PPI was evaluated for predicting several outcomes. Relying on its relation to the sympathetic tone, low PPI was able to predict hypotension during intermittent and continuous haemodialysis (22). A pre-dialysis PPI ≤ 1.8 can predict hypotension during dialysis with a positive predictive value of 80% and a negative predictive value of 100% (22). During weaning of mechanical ventilation, there is a usual increase in the cardiac output due to shifting the patient from positive to negative thoracic pressure (23). This increase in cardiac output is considered one of the predictors of successful weaning (23, 24). This fact might explain the recent finding of the ability of the PPI to predict successful weaning (25). Furthermore, weaning failure might be associated with stress and sympathetic overactivity, which contributed to the relation of the PPI and weaning outcome. Failure of the PPI to increase by 40% by the end of the spontaneous breathing trial can predict reintubation with a negative predictive value of 95% (25).

## Limitations

The use of PPI in clinical practice has some limitations. (1) PPI is characterized by skewness and a wide range of measurements among normal persons; therefore, it is better to evaluate its changes in comparison to the bassline readings from the same person. (2) Care should always be paid to the possibility of poor signals especially in cold extremities low temperature and high doses of vasopressors. (3) Being a ratio between the pulsatile and non-pulsatile portions of peripheral blood flow, the PPI is not feasible for use in patients who receive extra-corporeal membrane oxygenation. (4) Being affected by two variables, namely, the cardiac output and the autonomic activity, evaluation of the change in the PPI should be performed over short intervals where one of these two variables is relatively constant so that the PPI could be closely correlated to only one variable. However, even if it was affected by the two variables, the PPI could provide a good idea of patient prognosis because both variables affect the PPI in the same direction.

The utility of PPI in clinical practice is still a subject of ongoing research. Future studies are needed to evaluate the correlation between PPI and brain perfusion.

From the currently available evidence, we can conclude that PPI is an irreplaceable vital sign with many important uses, such as a prognostic marker in critically ill and surgical patients, guiding fluid and vasopressor management, assessing the success of weaning from mechanical ventilation, and can be used as an objective measure for the assessment of regional anesthesia and pain.

## Author Contributions

MME, MM, and RG contributed to the conception of the idea, literature search, collecting material, and drafting the manuscript. AH contributed to the conception of the idea, literature search, collecting materials, and drafting and revising the manuscript. All authors contributed to the article and approved the submitted version.

## Conflict of Interest

The authors declare that the research was conducted in the absence of any commercial or financial relationships that could be construed as a potential conflict of interest.

## Publisher's Note

All claims expressed in this article are solely those of the authors and do not necessarily represent those of their affiliated organizations, or those of the publisher, the editors and the reviewers. Any product that may be evaluated in this article, or claim that may be made by its manufacturer, is not guaranteed or endorsed by the publisher.
